# Preoperative predictors of chronic pain after laparoendoscopic groin hernia repair: A Swedish Hernia Registry study

**DOI:** 10.1007/s10029-025-03428-2

**Published:** 2025-10-18

**Authors:** Bengt Novik, Gabriel Sandblom, Anders Thorell

**Affiliations:** 1 Department of Clinical Sciences, Danderyd Hospital, Karolinska Institutet, Stockholm, Sweden; 2 Department of Clinical Science and Education, South Hospital, Karolinska Institutet, Stockholm, Sweden; 3 Department of Surgery, South Hospital, Stockholm, Sweden; 4 Department of Surgery and Anesthesiology, Ersta Hospital, Stockholm, Sweden

**Keywords:** Groin hernia, Inguinal hernia, Femoral hernia, Laparoendoscopic, Laparoscopic, Chronic postoperative inguinal pain, Patient-reported outcomes, Preoperative risk factors, Swedish Hernia Registry, Swedish Hernia Register

## Abstract

**Purpose:**

Chronic postoperative inguinal pain (CPIP) is a major adverse outcome of groin hernioplasty. Despite multiple CPIP studies, investigations involving unselected patients and sufficiently large cohorts to assess multiple predictors concurrently are still needed. This study evaluated the relative impact of preoperative predictors of CPIP after laparoendoscopic groin hernia repair. A secondary aim was to assess selection bias.

**Methods:**

This population-based cohort study included unilateral laparoendoscopic repairs from a prospective CPIP project within the Swedish Hernia Registry, where all groin hernia repairs recorded between September 2012 and December 2018 were surveyed at 1 year postoperatively.

Responses were analyzed using multivariable logistic regression to assess whether any of 15 predetermined preoperative candidate variables, including demographics, comorbidities, and hernia-related factors, were associated with CPIP.

**Results:**

Among 15 360 eligible patients, 10 525 (69%) responded, of whom 3 027 (29%) reported CPIP.

Seven preoperative factors significantly associated with CPIP:

• female sex (adjusted odds ratio [AOR] 1.15, 95% CI 1.03–1.28)

• younger age (< 45 vs. ≥ 65 years: AOR 1.36, 95% CI 1.25–1.49)

• BMI > 25 kg/m² (AOR 1.38, 95% CI 1.26–1.51)

• ASA grade > 1 (AOR 1.23, 95% CI 1.12–1.36)

• recurrent hernia (AOR 1.33, 95% CI 1.19–1.49)

• femoral hernia (vs. medial and/or lateral; AOR 1.20, 95% CI 1.002–1.43)

• small defects (AOR 1.18, 95% CI 1.06–1.30)

Response rates varied significantly across most variables, particularly age; non-respondents were on average 7 years younger, suggesting selection bias.

**Conclusion:**

The 7 identified preoperative predictors should be considered when designing and interpreting CPIP studies.

Selection bias was present, but likely less pronounced than in previous CPIP surveys.

Future research should incorporate preoperative pain assessment, improve response rates among younger patients, and conduct robust non-respondent analyses.

**Supplementary Information:**

The online version contains supplementary material available at 10.1007/s10029-025-03428-2.

## Introduction

Chronic postoperative inguinal pain (CPIP) is the foremost long-term complication after groin (inguinal and femoral) hernia repair. It is typically defined as pain persisting beyond the expected healing period of 3–6 months, with reported prevalence ranging from 0.7% to over 75%, depending on the definition used, measurement tool, and follow-up duration [1–4]. Symptoms vary from mild discomfort to debilitating pain, placing a substantial burden on both patients and society, given the high volume of groin hernia repairs. Despite various treatment strategies, high-quality evidence guiding optimal CPIP management remains limited, leaving many patients with persistent suffering that can last for years [5–8].

Interventions intended to reduce CPIP risk are challenging to evaluate scientifically, for several reasons. A common concern is the poor comparability of patient-reported outcome measurement tools, including hernia-specific questionnaires [9, 10]. Another key issue, which has received less attention in the design and interpretation of CPIP research, is the lack of consensus on which effect modifiers and confounders should be recorded and adjusted for.

Laparoendoscopic methods—primarily totally extraperitoneal (TEP) and transabdominal preperitoneal (TAPP) mesh repairs—have steadily gained preference over the past 3 decades. These approaches have been associated with a lower CPIP risk than open procedures [2, 11–19]. Nevertheless, a substantial proportion of patients undergoing laparoendoscopic repair still develop CPIP [20, 21]. Thus, comprehensive studies using robust real-world data to identify CPIP predictors in laparoendoscopic surgery are warranted [22].

Previous CPIP studies—mostly focused on open repairs—have identified several preoperative factors, including preoperative pain, female sex, younger age, higher American Society of Anesthesiologists (ASA) physical status grade, elevated body mass index (BMI), recurrent hernia, femoral hernia, and small hernia defect size [2, 3, 17, 20, 23–25]. However, many of these studies were statistically underpowered or included only a limited number of candidate variables, potentially resulting in imprecise multivariable adjustment or inflated risk estimates. The relative contributions of multiple preoperative risk factors remain unclear. Moreover, potential sources and effects of bias have rarely been adequately addressed.

The aim of this study was to identify and quantify preoperative predictors for CPIP 1 year after unilateral laparoendoscopic hernia repair. To this end, we investigated a broad set of potential risk factors, using over 6 years of detailed data from the Swedish Hernia Registry (SHR). Potential sources of bias and uncertainty were also critically assessed.

## Methods

The study was approved by the Regional Ethics Review Board in Stockholm, Sweden (EPN 2008/1082-31/2; amendments EPN 2014/2176-32 and EPN 2018/2050-32), and is registered at ClinicalTrials.gov (ID: NCT04838028). It adheres to the Strengthening the Reporting of Observational Studies in Epidemiology (STROBE) guidelines and the REporting of studies Conducted using Observational Routinely-collected health Data (RECORD) extension [26, 27].

### Study design and setting

Swedish healthcare is primarily tax-funded, with negligible patient co-payment, ensuring that surgery is accessible to virtually all residents.

This nationwide study included a consecutive, unselected cohort of patients who underwent laparoendoscopic groin hernia repairs over 76 months (> 6 years). Data were obtained from the SHR, a government-funded, non-profit registry that captures more than 95% of all groin hernia repairs in Sweden. The SHR provides systematic, continuously updated follow-up, refreshed weekly, until death, emigration, or patient opt-out [28]. Detailed descriptions of SHR data collection and validation processes have been published previously [29–33].

### Cohort

The study was based on a prospective follow-up project that included all groin hernia repairs registered in the SHR between September 1, 2012, and December 31, 2018. All surviving patients were invited to complete a brief, patient-reported outcome questionnaire 1 year postoperatively [34]. To assess CPIP, it included the first item of the validated Short-form Inguinal Pain Questionnaire (sf-IPQ) [35]. Nearly all returned questionnaires were received by the SHR 12–15 months postoperatively.

This analysis used a comprehensive SHR dataset containing detailed patient and surgical information from September 1, 2012, with follow-up data (questionnaire responses and reoperations) through November 6, 2020. Patients who underwent bilateral repairs were excluded, as the single questionnaire did not distinguish between left- and right-sided pain. Consequently, only unilateral repairs were included.

### Variables

The study endpoint, CPIP, was based on the patient’s reported maximum pain in the operated groin during the preceding week [35]. The 7-point Likert scale was dichotomized to classify outcomes as CPIP-positive or CPIP-negative (Table 1). A broader definition (CPIP-1) was used for the primary outcome to maximize statistical power, while a narrower definition (CPIP-2) served for sensitivity analysis.

**Table 1:** The first item of the Short-Form inguinal pain questionnaire (sf-IPQ)

Rate the worst pain you have felt in the operated groin during the recent week:	
1.	No pain
2.	Pain, could easily be ignored
3.	Pain, could not be ignored, but did not affect daily activities
4.	Pain, could not be ignored and interfered with concentration on chores/activities
5.	Pain, prevented most activities
6.	Pain, required bed rest
7.	Pain, prompted seeking immediate medical help

Primary outcome (**CPIP-1**) = Levels 3–7

Secondary outcome (**CPIP-2**) = Levels 4–7

#### Primary outcome (CPIP-1)

The primary outcome defined CPIP as any of the 5 highest pain levels on the sf-IPQ, starting with “*Pain that could not be ignored but did not affect daily activities*,” in line with some prior studies [30, 36–39]. The 2 lowest levels—“*No pain*” and “*Pain*,* could easily be ignored*”—were classified as CPIP-negative.

Additionally, patients who underwent reoperation within 15 months postoperatively were classified as CPIP-positive. This period comprised the postoperative interval during which most questionnaires were returned. This pragmatic decision assumed that virtually all such reoperations, including those for recurrence, were performed for painful conditions, thereby reducing underestimation of CPIP. It also addressed cases where questionnaires completed after a second operation failed to capture the index repair outcome. While this strategy may slightly overestimate CPIP incidence, we considered overestimation preferable to underestimation, given that most groin hernia patients experience some degree of preoperative pain [40].

Respondents were defined as patients who either completed the questionnaire and/or underwent new ipsilateral repair within 15 months. All others were classified as non-respondents. Patients reoperated for early complications (e.g., hematoma or infection) not requiring new hernioplasty were excluded due to insufficient procedural detail.

#### Secondary outcome (CPIP-2)

The secondary outcome used a stricter CPIP definition, requiring pain that at least “*interfered with concentration on chores/activities*.” Reoperations were disregarded in this definition. This narrower criterion aligns with most previous studies using the same sf-IPQ item [17, 30, 34, 41–44]. The two CPIP definitions were compared in a sensitivity analysis to assess the robustness of the findings.

#### Predictors of CPIP

Eighteen—15 preoperative and 3 intraoperative—candidate variables were evaluated as potential predictors (Tables 2 and 3).

Sex was treated as a binary biological variable. Age and BMI were recorded as continuous variables but categorized *post hoc* to enhance analysis and interpretation. Smoking was defined as current use at the time of surgery, including temporary perioperative abstinence. Comorbidities were not mandatory SHR fields and were Likely underreported. Bleeding diathesis and immunosuppression included both pathological and iatrogenic causes. Emergency repair was defined as surgery performed within 24 h of seeking acute care. Hernia anatomy and defect size were categorized according to the European Hernia Society (EHS) classification [3, 45].

In addition, 3 intraoperative variables were evaluated: repair type (TEP vs. TAPP), surgeon level, and surgical center.

### Data access and cleaning

The first author (BN) retrieved a complete, unrestricted, and unfiltered dataset from the SHR, including all recorded repairs and variables without prior selection or filtering. The data were manually reviewed for inconsistencies and implausible entries. Obvious errors were corrected when the true value could be confidently inferred; otherwise, values were recoded as missing.

### Statistical methods

Independent samples t-tests were used to compare baseline variables between respondents and non-respondents. For these comparisons, categorical variables with more than 2 levels were dichotomized.

The main analysis of CPIP risk factors employed multivariable logistic regression to estimate adjusted odds ratios (AORs) with 2-tailed 95% confidence intervals. All 18 candidate predicting variables were initially included, and backward stepwise selection was used to refine the model, retaining variables with *P* < 0.10. In the subsequent final model, statistical significance was set at *P* < 0.05.

Calculations were conducted using IBM^®^ SPSS^®^ Statistics, version 28 (IBM, Armonk, New York, USA).

## Results

### Participants and CPIP prevalence

During the inclusion period, the SHR recorded more than 100 000 hernia repairs in 87 668 patients [35].

**Fig. 1:**
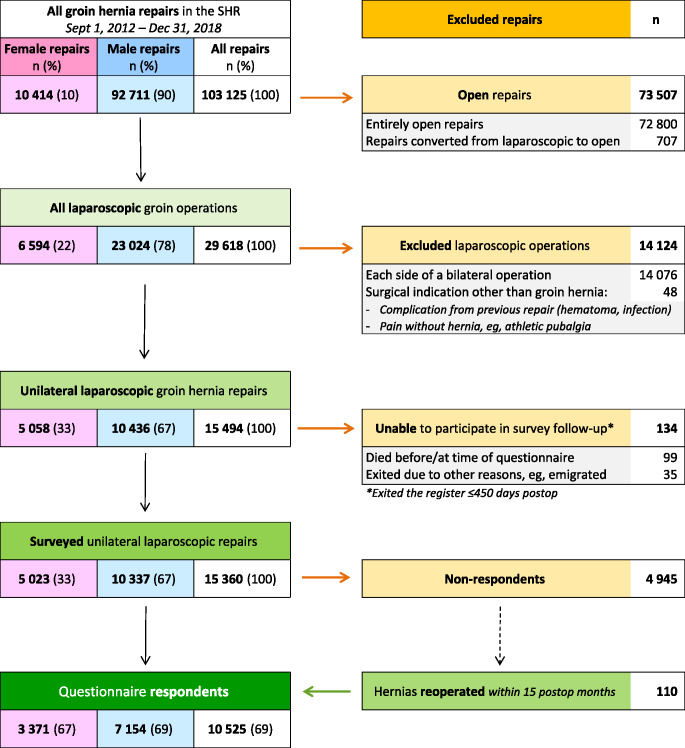
Flowchart of included and excluded repairs

Figure 1 shows the subsequent exclusions and the remaining cohort, stratified by sex. In almost all cases, the questionnaires returned were received by the SHR within 15 months.

A total of 82 centers contributed at least 1 repair that met the inclusion criteria, yielding 15 360 patients eligible for this study. Among these, 110 non-respondents who underwent subsequent ipsilateral repair within 15 months of surgery were reclassified as CPIP-positive respondents per study protocol and included in the main analysis. Including them, 10,525 (69%) were classified as questionnaire respondents and qualified for CPIP analysis.

Tables 2 and 3 compare the demographic and clinical features of respondents and non-respondents. Response rates varied significantly across 11 patient and surgical characteristics. Women, younger patients, those otherwise healthy (ASA 1), and smokers were less likely to respond. Lower response rates were also observed among patients who had primary repairs, emergency procedures, femoral hernias, small hernia defects, or treatment at low-volume surgical centers.

**Table 2:** Baseline patient characteristics, with respondent vs. non-respondent comparison

		All patients		Respondents		Non-respondents		
Variable	Category	*N*	(%)	*N*	(%)	*N*	(%)	*P*
	**All**	**15 360**	(100)	10 525	(**69**)	4 835	(**31**)	
Sex	Male	10 337	(67)	7 154	(68)	3 183	(66)	
	Female	5 023	(33)	3 371	(32)	1 652	(34)	**0.009**
Age (years)	Range, min ─ max	15 ─ 95		15 ─ 94		15 ─ 95		
	Mean (SD)	56.7 (16.2)		58.9 (15.1)		51.6 (17.2)		< **0.001**
BMI (kg/m^2^)	Range, min ─ max	14.5 ─ 60.5		14.9 ─ 60.5		14.5 ─ 54.6		
	Mean (SD)	24.8 (3.4)		24.8 (3.2)		25.0 (3.6)		**0.001**
ASA grade	ASA 1	8 271	(54)	5 498	(52)	2 773	(57)	
	ASA 2–4	7 089	(46)	5 027	(48)	2 062	(43)	<** 0.00**1
Smoking	No	9 146	(95)	6 095	(96)	3 051	(93)	
	Yes	452	(5)	238	(4)	214	(7)	< **0.001**
	*Missing data*	*5 762*		*4 192*		*1 570*		
Diabetes mellitus	No	9 311	(97)	6 132	(97)	3 179	(97)	
	Yes	288	(3.0)	202	(3.2)	86	(2.6)	0.12
	*Missing data*	*5 761*		*4 191*		*1 570*		
Collagen disorder	No	9 556	(99.6)	6 304	(99.5)	3 252	(99.6)	
	Yes	43	(0.4)	30	(0.5)	13	(0.4)	0.59
	*Missing data*	*5 761*		*4 191*		*1 570*		
COPD/asthma	No	9 221	(96)	6 095	(96)	3 126	(96)	
	Yes	378	(3.9)	239	(3.8)	139	(4.4)	0.26
	*Missing data*	*5 761*		*4 191*		*1 570*		
Bleeding diathesis	No	9 172	(96)	6 034	(95)	3 138	(96)	
	Yes	427	(4.4)	300	(4.7)	127	(3.9)	**0.050**
	*Missing data*	*5 761*		*4 191*		*1 570*		
Immuno-suppression	No	9 489	(99)	6 258	(99)	3 231	(99)	
	Yes	110	(1.1)	76	(1.2)	34	(1.0)	0.48
	*Missing data*	*5 761*		*4 191*		*1 570*		

In parentheses are percentages, except for Age and BMI where SD = standard deviation

BMI = Body mass index

ASA = American Society of Anesthesiologists

COPD = Chronic obstructive pulmonary disease

*P*-values (2-tailed, α = 0.05) regard the difference in distribution between respondents and non-respondents within each variable category

Missing data were excluded from each significance analysis

**Table 3:** Baseline hernia and surgery data, with respondent vs. non-respondent comparison

			All patients		Respondents		Non-respondents		
Variable	Category		*N*	(%)	*N*	(%)	*N*	(%)	*P*
All			15 360	(100)	10 525	(**69**)	4 835	(**31**)	
Indication		Primary hernia	12 036	(78)	8 122	(77)	3 914	(81)	
		Recurrent hernia	3 324	(22)	2 403	(23)	921	(19)	< **0.001**
Urgency		Elective repair	14 966	(97)	10 287	(98)	4 679	(97)	
		Emergency	394	(2.6)	238	(2.3)	156	(3.2)	< **0.001**
Laterality		Right groin	8 950	(58)	6 146	(58)	2 804	(58)	
		Left groin	6 410	(42)	4 379	(42)	2 031	(42)	0.64
Hernia anatomy (EHS classification)		Inguinal	13 691	(93)	9 451	(93)	4 240	(91)	
		Femoral only	1 078	(7)	676	(7)	401	(9)	<** 0.00**1
		*Other/Unspecified*	*592*		*398*		*194*		
Hernia defect size (EHS classification)		I (small)	4 289	(28)	2 783	(26)	1 506	(31)	
		II/III (medium/large)	11 063	(72)	7 734	(74)	3 329	(69)	<** 0.001**
		Missing data	8		8		0		
Type of repair		TEP	12 884	(84)	8 864	(85)	4 020	(84)	
		TAPP	2 372	(16)	1 589	(15)	783	(16)	0.085
		*Other/Unspecified*	*104*		*72*		*32*		
Surgeon level		Resident	401	(2.7)	282	(2.7)	119	(2.5)	
		Certified surgeon	14 627	(97)	10 073	(97)	4 554	(97)	0.53
		*Missing data*	*332*		*170*		*162*		
Surgical center*		High volume	11 315	(74)	7 900	(75)	3 415	(71)	
		Low volume	4 045	(26)	2 625	(25)	1 420	(29)	<** 0.001**

EHS = European Hernia Society. Inguinal = Lateral and/or Medial

Defect size (Ø): I < 1.5 cm); II = 1.5–3 cm; III > 3 cm

TEP = Totally extra-peritoneal repair. TAPP = Trans-abdominal preperitoneal repair

*Surgical center:

High volume = A center that conducted > 200 laparoendoscopic groin hernia repairs during the inclusion period, also comprising those—mainly bilateral—that were excluded in this study.

Low volume = All other centers

*P*-values (2-tailed, α = 0.05) regard the difference in distribution between responders and non-responders within each variable category

Missing data were excluded from the significance analyses

**Fig. 2:**
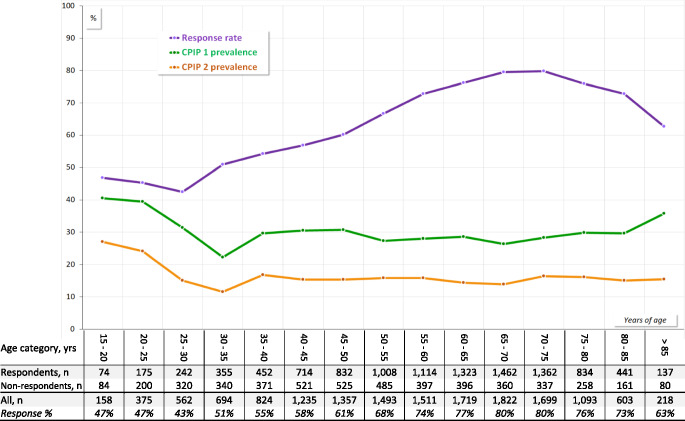
Response rate and CPIP prevalences, by age category. Overall questionnaire response rate = 68%. CPIP = Chronic postoperative inguinal pain prevalence at 1 year. CPIP-1 (primary definition) overall prevalence = 29%. CPIP-2 (secondary definition) overall prevalence = 15%

Figure 2 illustrates age-related response variability (top curve), and CPIP prevalence using both definitions: 3 027 of 10 525 (29%) for the primary definition (middle curve), and 1 614 of 10 415 (15%) for the secondary definition (bottom curve).

### Main analysis

An initial stepwise selection process eliminated 8 preoperative variables (smoking, diabetes, chronic obstructive pulmonary disease/asthma, bleeding diathesis, immunosuppression, collagen disorder, urgency, and groin side), and 1 intraoperative variable (surgeon level), none of which met the *P* < 0.10 inclusion threshold (Table 4).

**Table 4:** Insignificant CPIP predictor candidates, stepwise eliminated

Variable	Category	*N*	%	AOR	95% CI	*P*
All survey respondents		**10 525**	(100%)			
Bleeding diathesis	No bleeding diathesis	6 034	(95%)	**1**	Reference	
	Bleeding diathesis	300	(5%)	**1.1**	0.87 ─ 1.5	0.38
	**Sum**	**6 334**	(61%)			
Immuno- suppression	No immuno-suppression	6 258	(99%)	**1**	Reference	
	Immuno-suppression	76	(1%)	**0.90**	0.54 ─ 1.5	0.67
	**Sum**	**6 334**	(61%)			
Collagen disease	No collagen disease	6 304	(99,5%)	**1**	Reference	
	Collagen disease	30	(0,5%)	**1.1**	0.50 ─ 2.3	0.86
	**Sum**	**6 334**	(60%)			
COPD/asthma	No COPD/asthma	6 095	(96%)	**1**	Reference	
	COPD/asthma	239	(4%)	**1.0**	0.78 ─ 1.4	0.77
	**Sum**	**6 334**	(60%)			
Diabetes mellitus	No diabetes	6 132	(97%)	**1**	Reference	
	Diabetes	202	(3%)	**0.79**	0.57 ─ 1.1	0.16
	**Sum**	**6 334**	(60%)			
Smoking	No smoker	6 095	(96%)	**1**	Reference	
	Smoker	238	(4%)	**1.2**	0.90 ─ 1.6	0.21
	**Sum**	**6 333**	(60%)			
Laterality	Right groin	6 146	(58%)	**1**	Reference	
	Left groin	4 379	(42%)	**1.0**	0.93 ─ 1.1	0.72
	**Sum**	**10 525**	(100%)			
Emergency	Elective	10 287	(98%)	**1**	Reference	
	Emergency	238	((2%)	**1.2**	0.89 ─ 1.6	0.24
	**Sum**	**10 525**	(100%)			
Surgeon level	Resident	282	(3%)	**1**	Reference	
	Certified surgeon	10 073	(97%)	**0.85**	0.65 ─ 1.1	0.20
	**Sum**	**10 355**	(98%)			

CPIP = Chronic postoperative inguinal pain

AOR = adjusted odds ratio of experiencing CPIP, and/or having been reoperated at 15 months postop.

COPD = chronic obstructive pulmonary disease.

The final model included 7 preoperative and 2 intraoperative risk factors (Table 5).

Female sex was associated with modestly increased risk.

**Table 5:** Main analysis: Proportionate risks of chronic inguinal pain at 1 year postoperatively

Variable	Category	*N*	(%)	AOR	95% CI	*P*
Sex	Male	7 154	(68)	**1**	reference	
	Female	3 371	(32)	**1.15**	1.03 ─ 1.28	**0.010**
Age (years)	15–30	491	(4.7)	**1.94**	1.54 ─ 2.44	<** 0.001**
	30–45	1 521	(14)	**1.28**	1.08 ─ 1.52	**0.004**
	45–55	1 840	(17)	**1.25**	1.07 ─ 1.47	**0.006**
	55–65	2 437	(23)	**1.17**	1.01 ─ 1.36	**0.034**
	65–70	1 462	(14)	**1**	reference	
	70–75	1 362	(13)	**1.06**	0.90 ─ 1.26	0.48
	> 75	1 412	(13)	**1.11**	0.94 ─ 1.31	0.24
BMI (kg/m^2^)	< 20	500	(4.8)	**1.14**	0.92 ─ 1.39	0.23
	20–25	5 255	(50)	**1**	reference	
	25–30	3 757	(36)	**1.36**	1.24 ─ 1.50	<** 0.001**
	30–35	508	(4.8)	**1.57**	1.29 ─ 1.90	<** 0.001**
	> 35	66	(0.6)	**1.86**	1.13 ─ 3.07	**0.015**
	*Missing value*	*439*	*(4.2)*			
ASA grade	ASA 1	5 498	(52)	**1**	reference	
	ASA 2	4 266	(41)	**1.19**	1.07 ─ 1.31	<** 0.001**
	ASA 3	750	(7.1)	**1.51**	1.26 ─ 1.81	<** 0.001**
	ASA 4	11	(0.1)	**2.49**	0.75 ─ 8.29	0.14
Indication	Primary hernia	8 122	(77)	**1**	reference	
	Recurrent hernia	2 403	(23)	**1.33**	1.19 ─ 1.49	< **0.001**
Hernia anatomy (EHS classification)	Inguinal^**a**^	9 451	(90)	**1**	reference	
	Femoral	676	(6.4)	**1.20**	1.002 ─ 1.43	**0.047**
	Femoral combinations^b^	327	(3.1)	**1.16**	0.91 ─ 1.48	0.23
	*Other/Unspecified*	*71*	*(0.7)*			
Hernia defect size (EHS classification)^c^	I – small Ø	2 783	(26)	**1.18**	1.06 ─ 1.31	**0.002**
	II/III – medium or large Ø	7 734	(73)	**1**	reference	
	Missing value	8	(0.1)			
Repair method	TEP	8 864	(84)	**1**	reference	
	TAPP	1 589	(15)	**1.16**	1.03 ─ 1.31	**0.012**
	*Other/Unspecified*	*72*	*(0.7)*			
Surgical center	All 82 centers	10 525	(100)	**1.004**	1.001 ─ 1.007	**0.008**

AOR Multivariable adjusted odds ratio

BMI =Body mass index. ASA = American Society of Anesthesiologists. EHS = European Hernia Society

^a^Inguinal: Lateral, Medial, or Lateral + Medial

^b^Femoral combinations: Lateral + Femoral, Medial + Femoral, or Lateral + Medial + Femoral

^c^Hernia defect size: I < 1.5 cm; II = 1.5–3 cm; III > 3 cm

TEP = Totally extra-peritoneal repair. TAPP = Trans-abdominal preperitoneal repair

Age, treated as a continuous variable, showed a weak inverse correlation with likelihood of CPIP (AOR 0.990, CI 0.988–0.993; *P* < 0.001). Due to non-linearity, age was analyzed *post hoc* in categories. Compared to patients 65 years or older, those younger than 45 years (AOR 1.36, CI 1.25–1.49, *P* < 0.001), and 45–64 years (AOR 1.34, CI 1.08–1.65, *P* = 0.008), were associated with higher CPIP risk. For more detailed analysis, the final model comprised 7 discrete age categories (Table 5).

Higher ASA grade and increasing BMI above normal weight (> 25 kg/m^2^), were each associated with progressively higher CPIP risks.

Recurrence repairs comprised 3 324 (22%) of the index procedures and were linked to an increased CPIP risk. Some may argue that including recurrent repairs would confound the results, and thereby disturb interpretation. To address this, we added a Supplementary Table where the outcomes are stratified by primary vs. recurrent repairs, demonstrating only minor differences in AORs (except for categories with very low numbers).

Given the similar risks associated with lateral (AOR 1.01, CI 0.91–1.13) and combined medial-lateral hernias (AOR 0.99, CI 0.80–1.22), these categories were merged with medial hernias as the reference group. Using this combined group as the reference, femoral hernias were associated with elevated risk.

Similarly, medium-size (EHS II) defects (AOR 0.99, CI 0.86–1.13) and large (EHS III) defects had comparable risk and were therefore merged for analysis. In comparison, small (EHS I) defects were associated with an increased CPIP risk.

### Sensitivity analysis

Applying the stricter CPIP-2 definition (secondary outcome) reduced the overall prevalence by nearly half, attenuating statistical power. Nonetheless, the relative risk pattern remained consistent, as illustrated in Table 6 and the lower two curves of Fig. 2. No major discrepancies were observed between the main and sensitivity analyses.

**Table 6:** Sensitivity analysis: secondary outcome (CPIP-2) *versus* primary outcome (CPIP-1)

				CPIP-2			CPIP-1	CPIP-2 vs. CPIP-1	
Variable	Category	*N*	%	AOR 2	95% CI	*P*	AOR 1	AOR 2 * divided by* AOR 1	AOR 2 *minus* AOR 1
Sex	Male	7 064	68%	**1**	reference				
	Female	3 351	32%	**1.14**	0.997─1.30	0.055	**1.15**	1.0	0.0
Age (years)	15–30	483	5%	**1.91**	1.44─2.54	< **0.001**	**1.95**	1.0	0.0
	30–45	1 502	14%	**1.27**	1.02─1.57	**0.034**	**1.29**	1.0	0.0
	45–55	1 813	17%	**1.29**	1.05─1.58	**0.015**	**1.25**	1.0	0.0
	55–65	2 410	23%	**1.18**	0.975─1.42	0.090	**1.17**	1.0	0.0
	65–70	1 449	14%	**1**	reference				
	70–75	1 355	13%	**1.16**	0.94─1.43	0.16	**1.06**	1.1	0.1
	> 75	1 403	13%	**1.03**	0.83─1.27	0.80	**1.11**	0.9	−0.1
BMI (kg/m^2^)	< 20	495	5%	**1.27**	0.99─1.63	0.055	**1.13**	1.1	0.1
	20–25	5 225	50%	**1**	reference				
	25–30	3 702	36%	**1.25**	1.11─1.41	<** 0.001**	**1.36**	0.9	−0.1
	30–35	499	5%	**1.52**	1.20─1.92	< **0.001**	**1.57**	1.0	−0.1
	> 35	65	0.6%	**1.62**	0.91─2.87	0.10	**1.86**	0.9	−0.2
	*Missing value*	429	4%	**1.33**			**1.35**	1.0	0.0
ASA grade	ASA 1	5 436	52%	**1**	reference				
	ASA 2	4 223	41%	**1.24**	1.09─1.41	< **0.001**	**1.19**	1.0	0.1
	ASA 3	745	7%	**1.71**	1.38─2.12	< **0.001**	**1.51**	1.1	0.2
	ASA 4	11	0.1%	**2.50**	0.65─9.58	0.18	**2.42**	1.0	0.1
Indication	Primary hernia	8 053	77%	**1**	reference				
	Recurrent hernia	2 362	23%	**1.25**	1.09─1.44	**0.001**	**1.32**	0.9	−0.1
Hernia anatomy	Medial and/or Lateral	2 662	26%	**1**	reference				
	Femoral	672	6%	**1.15**	0.92─1.43	0.21	**1.20**	1.0	0.0
	Femoral combinations	205	2%	**1.30**	0.98─1.74	0.072	**1.16**	1.1	0.1
	*Other or Missing value*	*69*	*0.7%*	***2.11***	1.24─3.58	**0.006**	**1.96**	1.1	0.1
Hernia defect Ø	EHS I: < 1.5 cm	2 758	26%	**1.26**	1.11─1.42	< **0.001**	**1.18**	1.1	0.1
	EHS II-III: ≥ 1.5 cm	7 649	73%	**1**	reference				
	*Missing value*	8	0.1%	**1.54**			**1.21**	1.3	0.3
Surgical method	TEP	8 766	84%	**1**	reference				
	TAPP	1 580	15%	**1.13**	0.98─1.31	0.092	**1.16**	1.0	0.0
	*Other or Unspecified*	*69*	*0.7%*	***1.34***			***1.16***	*1.2*	*0.2*
Surgical center		10 415	100%	**1.002**	0.998─1.005	0.38	**1.004**	1.0	0.0

AOR 2 = Adjusted odds ratio of CPIP 2 (secondary outcome)

AOR 1 = Adjusted odds ratio of CPIP 1 (primary outcome)

EHS = European Hernia Society

## Discussion

This is the largest study to date on preoperative predictors of CPIP following consecutive, unselected, unilateral laparoendoscopic groin hernia repairs, with surgeons also unselected and without influence on follow-up. The use of an extensive, population-based cohort enhanced statistical power, improved precision, and strengthened generalizability. We analyzed data from 15 360 unilateral laparoendoscopic groin hernia repairs recorded in the SHR over a period exceeding 6 years. The postoperative survey after 1 year reached a 69% response rate.

Three key findings emerged. First, 7 preoperative predictors independently associated with CPIP: female sex, younger age, ASA > 1, increasing BMI above normal, recurrence repair, femoral hernia, and small hernia defects. Second, despite the broad primary CPIP definition, sensitivity analysis using a stricter threshold yielded consistent odds ratios, indicating that risk estimates remain stable across different definitions. Third, response rates varied significantly across most variables—especially age—implying potential non-response bias, an issue rarely addressed in prior studies.

The identified risk factors align with previous CPIP studies— primarily on open repairs—while refining risk assessment by quantifying the multivariable-adjusted contribution of each risk factor in the context of laparoendoscopic repair. Our findings suggest that preoperative CPIP predictors are largely consistent across surgical approaches.

### Strengths

The SHR, alongside the Danish Hernia Database, are worldwide the only registries of groin hernia repair with true nationwide coverage and lifelong individual follow-up, minimizing selection and attrition bias [31]. Sweden’s healthcare system ensures all residents have access to hernia repair, allowing the SHR data to reflect the entire adult population. The cohort analyzed comprised at least 95% of all unilateral laparoendoscopic groin hernia repairs performed in Sweden during the inclusion period. Annual validation of SHR data mitigates misclassification and supports internal validity [28]. The use of a validated patient-reported outcome measure, a fixed 1-year follow-up, and high response rate further enhance the robustness and generalizability of the results. A major advantage in this study was that the questionnaires were mailed, collected, registered, and assessed independently of the surgical centers. This minimized observer and input bias, thereby enhancing validity [31]. Additionally, the retrieved study database underwent thorough scrutiny to minimize data entry errors.

### Limitations

Despite these advantages, selection bias cannot be entirely excluded. The overall response rate (69%) was higher than in comparable registry-based studies [24, 38]. Yet, non-response bias remains a concern. Response rates varied across most variables. Younger and very elderly patients, women, those with higher BMI, and ASA 1, smokers, primary hernia, emergency repair, femoral hernia, and small (EHS I) defects were less likely to respond. While age- and sex-related response biases have been noted in a few previous studies, they are often only briefly mentioned [46, 47]. In a prior SHR study based on a different subset of the same larger cohort from which the present sample was drawn, telephone interviews with a random test sample of 119/8 005 (1.5%) non-respondents revealed a sixfold lower CPIP prevalence compared to respondents [34]. If generalizable, this suggests that both the present and previously reported risk estimates in, e.g., younger patients, may be exaggerated.

Although we adjusted for multiple covariates, some potentially relevant predictors—such as preoperative pain, socioeconomic status, and psychiatric history—were not recorded in the SHR during the study period [5, 12, 48]. While these factors may influence CPIP risk, they are unlikely to systematically vary across the included 18 variables in a way that would materially alter the findings.

It is important to differentiate selection bias inherent to study design from the clinical selection that occurs in routine practice. This distinction is particularly relevant when interpreting results related to preoperative groin pain, BMI, and ASA. Preoperative groin pain, a known risk factor for CPIP, is also a primary indication for hernia repair [2, 3, 5, 12, 40, 46, 49]. While hernia surgery aims to alleviate discomfort, it may not always accomplish that and can sometimes even inflict new pain [50]. Because preoperative symptoms were not recorded, distinguishing between pre-existing and surgery-induced pain was not feasible [51]. However, we presume that preoperative pain prevalence was evenly distributed across most subgroups, and therefore minimally impacted the estimated AORs, except for categories where the level of preoperative pain could have been a major determinant when selecting patients for surgery. Since groin hernias are rarely incapacitating or life-threatening, surgical indications are typically relative and based on shared decision-making. For example, obese or frail individuals face increased anesthetic and surgical risks [52–55]. Among such patients, physicians may be more likely to recommend conservative management for those with negligible discomfort and offer surgery to those with more intense pain [3, 50]. Consequently, CPIP estimates for overweight, obese, and high-ASA patients may potentially be overestimated in non-randomized studies, warranting cautious interpretation of CPIP risk in these categories.

In contrast to many healthcare systems where residence, ethnicity, or socioeconomic status influence access to surgical care, Sweden’s universal healthcare minimizes such disparities. Accordingly, these variables are unlikely to confound the findings, and are not collected in the SHR.

### Assessed variables

The seemingly high CPIP prevalence in this study should not be interpreted as inherent to the laparoendoscopic approach but rather reflects the chosen CPIP definition and its purpose. Multivariable models with numerous predictors can result in small subgroups and an increased risk of type II errors. To maximize power, we used a broad CPIP definition, yielding a 29% prevalence at 1 year. While about half of these cases involved fairly mild pain (IPQ level 3), the primary focus was not on prevalence but on relative differences in risk, expressed as odds ratios. Sensitivity analysis using a stricter CPIP definition confirmed the robustness of these risk estimates, although confidence intervals widened due to reduced statistical power.

The results confirm previous observations that women are at higher CPIP risk, extending this finding to laparoendoscopic repair [12, 17, 30]. This aligns with modern pain research, which highlights sex-related biological differences in pain perception. Historically, medical and biological research has been male-centric—focusing on male cells, animals, and humans— leaving female pain mechanisms underexplored. However, contemporary studies have established that female sex per se is an independent risk factor for chronic pain in general, likely due to genetic and biological influences [56, 57]. In this context, the modest increase in CPIP risk among women (AOR 1.15) is perhaps within or even below expected ranges, and not implying inferior surgical care.

The finding that younger age was associated with higher CPIP risk corroborates several previous studies [2, 3, 12, 20, 24, 25, 30, 46]. However, age also influenced response rates, potentially confounding the results—an issue rarely addressed in CPIP research. In contrast, a Dutch TEP study with near-complete follow-up at 1 year found no significant CPIP difference between young (18–30 years) vs. older (≥ 31 years) patients [49]. Moreover, we used an IPQ item, which—like many patient-reported outcome measures—assesses pain severity by using limitations in physical activity as proxy. Aasvang and Kehlet hypothesized that interpretations of “daily activities” vary by age. Younger individuals, engaging in more strenuous activities, may experience greater functional impairment from equivalent pain [2]. The above reflections imply a need for cautious interpretation of CPIP risk in younger populations.

The strong positive association between higher BMI and CPIP is consistent with previous research [20, 24, 25, 30]. How BMI affects CPIP risk may be multi-factorial. Adiposity increases surgical complexity and chances of complications [53–55]. Reasonably, an elevated CPIP risk follows. Conversely, patient selection may exaggerate the CPIP risk in overweight and obese individuals. Despite a higher hernia and recurrence risk, obese individuals are under-represented among surgical patients [58]. Excess subcutaneous inguinal fat can obscure symptoms and delay diagnosis of hernia [53, 54]. In addition, surgeons may be more reluctant to recommend repair for obese individuals with mild symptoms, due to the added surgical challenge [53]. Consequently, obese patients who undergo surgery likely have more severe preoperative symptoms on average than their non-operated counterparts, which introduces confounding by indication.

The high percentage (22%) of index repairs being recurrent reflects international guidelines recommending laparoscopic reoperation after open repair [3]. Recurrent repairs were associated with increased CPIP risk, which is reasonable and consistent with previous studies [2, 5, 25, 46].

Finally, small defects (EHS I) were associated with higher CPIP risk, mirroring reports from the *Herniamed* registry [20, 24]. However, patients with small hernias are more likely to undergo surgery if they experience pain, whereas those with minimal or no discomfort may not seek medical attention until the defect enlarges to an EHS II or III [50]. This selection effect may, at least partially, explain the observed association

Although surgical method and center volume influenced CPIP risk, these variables were primarily included to optimize model adjustment and are therefore beyond the scope of this discussion.

## Conclusions

In this large nationwide cohort, 7 key preoperative predictors of CPIP after laparoscopic unilateral groin hernia repair were identified and their relative impact quantified. The study also highlights selection bias as a major but under-recognized challenge in CPIP research. These real-world data may help refine future study protocols and improve outcome interpretation.

Although most of the identified predictors cannot be modified, the findings may enhance individualized patient counseling by helping to identify and prepare patients at increased CPIP risk.

Future studies should incorporate strategies to mitigate selection bias, including preoperative pain assessment, efforts to improve response rates among younger patients, and robust non-respondent analyses.

The findings from this study will inform the next phase of our research evaluating CPIP risks according to various mesh and fixation combinations.

## Electronic supplementary material

Below is the link to the electronic supplementary material.

Supplementary file 1 (PDF 208KB)

## Data Availability

The dataset analyzed in the current study was retrieved from the Swedish Hernia Registry, is not publicly available and cannot be requested from the authors. To access the data, a separate application must be made to the Registry.
