# Supplementary material for: Preoperative predictors of chronic pain after laparoendoscopic groin hernia repair: A Swedish Hernia Registry study
Source: Hernia. 2025 Oct 18;29(1):303. doi: 10.1007/s10029-025-03428-2 (PMC12534285; doi:10.1007/s10029-025-03428-2)
Supplement: Supplementary file 1 — (PDF 208KB) [file 10029_2025_3428_MOESM1_ESM.pdf]

Supplementary table: Relative risk of chronic inguinal pain at 1 year postoperatively
Stratified by primary and recurrent hernia repair

PRIMARY Hernia Repair					
**Variable**	**N**	***(%)***	**AOR**	***CI 95%***	**P**
**Sex**					
Male	4 886	*60%*	**1**	*reference*	
Female	3 236	*40%*	**1.11**	*0.996 - 1.2*	0.058
	**8 122**	*100%*			
**Age** *(years)*					
15 - 30	465	*5.7%*	**1.88**	*1.5 - 2.4*	<0.001
30 - 45	1 365	*17%*	**1.31**	*1.08 - 1.6*	0.007
45 - 55	1 518	*19%*	**1.26**	*1.04 - 1.5*	0.016
55 - 65	1 866	*23%*	**1.15**	*0.96 - 1.4*	0.12
65 - 70	1 026	*13%*	**1**	*reference*	
70 - 75	955	*12%*	**1.17**	*0.96 - 1.4*	0.13
> 75	927	*11%*	**1.25**	*1.02 - 1.5*	0.035
**BMI** *(kg/m²)*					
< 20	445	*5.5%*	**1.09**	*0.88 - 1.4*	0.44
20 - 25	4 179	*51%*	**1**	*reference*	
25 - 30	2 732	*34%*	**1.33**	*1.2 - 1.5*	<0.001
30 - 35	370	*4.6%*	**1.64**	*1.3 - 2.1*	<0.001
> 35	53	*0.7%*	**1.49**	*0.84 - 2.6*	0.17
Missing/Improbable value	343	*4.2%*			
> 30	423	*5.2%*	**1.62**	*1.3 - 2.0*	<0.001
**ASA**					
ASA 1	4 596	*57%*	**1**	*reference*	
ASA 2	3 036	*37%*	**1.18**	*1.05 - 1.3*	0.006
ASA 3	484	*6.0%*	**1.39**	*1.11 - 1.7*	0.004
ASA 4	6	*0.1%*	**0.63**	*0.07 - 5.5*	0.67
ASA 3-4	490	*6.0%*	**1.37**	*1.1 - 1.7*	0.005
**Hernia Anatomy**					
Inguinal	7 208	*89%*	**1**	*reference*	
Femoral	590	*7.3%*	**1.21**	*0.999 - 1.5*	0.051
Femoral combinations	264	*3.3%*	**1.19**	*0.91 - 1.6*	0.21
Other/Missing value	60	*0.7%*			
**Hernia Defect Ø**					
EHS I	2 278	*28%*	**1.19**	*1.06 - 1.34*	0.003
EHS II-III	5 844	*72%*	**1**	*reference*	
Missing value	0				
**Repair Method**					
TEP	6 971	*86%*	**1**	*reference*	
TAPP	1 109	*14%*	**1.13**	*0.98 - 1.31*	0.084
MIS, other/unspecified	42	*0.5%*			
**Surgical Center**	**8 122**	*100%*	**1.005**	*1.002 - 1.008*	0.002

Note: Categories with N = very low → unreliable AORs

RECURRENT Hernia Repair					
**Variable**	**N**	***(%)***	**AOR**	***CI 95%***	**P**
**Sex**					
Male	2 268	*94%*	**1**	*reference*	
Female	135	*6%*	**1.40**	*0.94 - 2.1*	0.098
	**2 403**	*100%*			
**Age** *(years)*					
15 - 30	26	*1.1%*	**3.96**	*1.7 - 9.1*	0.001
30 - 45	156	*6.5%*	**1.25**	*0.83 - 1.9*	0.28
45 - 55	322	*13%*	**1.26**	*0.92 - 1.7*	0.15
55 - 65	571	*24%*	**1.26**	*0.96 - 1.6*	0.10
65 - 70	436	*18%*	**1**	*reference*	
70 - 75	407	*17%*	**0.87**	*0.65 - 1.2*	0.37
> 75	485	*20%*	**0.92**	*0.69 - 1.2*	0.57
**BMI** *(kg/m²)*					
< 20	55	*2.3%*	**1.36**	*0.76 - 2.4*	0.30
20 - 25	1 076	*45%*	**1**	*reference*	
25 - 30	1 025	*43%*	**1.46**	*1.21 - 1.8*	<0.001
30 - 35	138	*5.7%*	**1.41**	*0.96 - 2.1*	0.080
> 35	13	*0.5%*	**5.09**	*1.34 - 19.3*	0.017
Missing/Improbable value	96	*4.0%*			
> 30	151	*6.3%*	**1.55**	*1.08 - 2.2*	0.019
**ASA**					
ASA 1	902	*38%*	**1**	*reference*	
ASA 2	1 230	*51%*	**1.22**	*0.99 - 1.5*	0.060
ASA 3	266	*11%*	**1.72**	*1.3 - 2.4*	<0.001
ASA 4	5	*0.1%*	**11.8**	*1.3 - 110*	0.030
ASA 3-4	271	*11%*	**1.86**	*1.4 - 2.5*	<0.001
**Hernia Anatomy**					
Inguinal	2 243	*93%*	**1**	*reference*	
Femoral	86	*3.6%*	**1.11**	*0.678 - 1.8*	0.68
Femoral combinations	63	*2.6%*	**1.02**	*0.59 - 1.8*	0.94
Other/Missing value	11	*0.5%*			
**Hernia Defect Ø**					
EHS I	505	*21%*	**1.15**	*0.92 - 1.42*	0.21
EHS II-III	1 890	*79%*	**1**	*reference*	
Missing value	8	*0.3%*			
**Repair Method**					
TEP	1 893	*79%*	**1**	*reference*	
TAPP	480	*20%*	**1.26**	*1.02 - 1.57*	0.035
MIS, other/unspecified	30	*1.2%*			
**Surgical Center**	**2 403**	*100%*	**1.00**	*0.995 - 1.006*	0.93

AOR = Multivariable adjusted odds ratio

BMI = Body mass index

ASA = American Society of Anesthesiologists' risk score

TEP = Totally extra-peritoneal repair

TAPP = Trans-abdominal preperitoneal repair

MIS = Minimally invasive surgery, eg, laparoscopy

Inguinal =
Lateral, *or* Medial, *or* Lateral/Medial

Femoral combinations =
Lateral/ Femoral, *or* Medial/ Femoral, *or* Lateral/ Medial/ Femoral

EHS = European Hernia Society

Hernia defect Ø: **I** < 1.5cm; **II** = 1.5-3 cm; **III** > 3cm
